# Complementary supramolecular drug associates in perfecting the multidrug therapy against multidrug resistant bacteria

**DOI:** 10.3389/fimmu.2024.1352483

**Published:** 2024-02-13

**Authors:** Pathik Sahoo

**Affiliations:** ^1^ International Center for Materials and Nanoarchitectronics (MANA), Research Center for Advanced Measurement and Characterization (RCAMC), National Institute for Materials Science, Tsukuba, Japan; ^2^ Foundation of Physics Research Center (FoPRC), Celico, Italy

**Keywords:** binary antibiotic systems, cell membrane permeability, complementary drugs, complementary multidrug cocrystal, crystal engineering, efflux pump, supramolecular synthon

## Abstract

The inappropriate and inconsistent use of antibiotics in combating multidrug-resistant bacteria exacerbates their drug resistance through a few distinct pathways. Firstly, these bacteria can accumulate multiple genes, each conferring resistance to a specific drug, within a single cell. This accumulation usually takes place on resistance plasmids (R). Secondly, multidrug resistance can arise from the heightened expression of genes encoding multidrug efflux pumps, which expel a broad spectrum of drugs from the bacterial cells. Additionally, bacteria can also eliminate or destroy antibiotic molecules by modifying enzymes or cell walls and removing porins. A significant limitation of traditional multidrug therapy lies in its inability to guarantee the simultaneous delivery of various drug molecules to a specific bacterial cell, thereby fostering incremental drug resistance in either of these paths. Consequently, this approach prolongs the treatment duration. Rather than using a biologically unimportant coformer in forming cocrystals, another drug molecule can be selected either for protecting another drug molecule or, can be selected for its complementary activities to kill a bacteria cell synergistically. The development of a multidrug cocrystal not only improves tabletability and plasticity but also enables the simultaneous delivery of multiple drugs to a specific bacterial cell, philosophically perfecting multidrug therapy. By adhering to the fundamental tenets of multidrug therapy, the synergistic effects of these drug molecules can effectively eradicate bacteria, even before they have the chance to develop resistance. This approach has the potential to shorten treatment periods, reduce costs, and mitigate drug resistance. Herein, four hypotheses are presented to create complementary drug cocrystals capable of simultaneously reaching bacterial cells, effectively destroying them before multidrug resistance can develop. The ongoing surge in the development of novel drugs provides another opportunity in the fight against bacteria that are constantly gaining resistance to existing treatments. This endeavour holds the potential to combat a wide array of multidrug-resistant bacteria.

## 1 Introduction

The ongoing battle between bacteria and the constant emergence of new drugs contributes to the rising issue of drug resistance, a problem exacerbated by the unregulated and irresponsible use of antibiotics. This has created an era where bacteria have gained immunity against multiple drugs. The consequences of this widespread antimicrobial resistance are alarming, with an estimated 4.95 million lives lost globally in 2019 due to bacterial resistance against 88 specific pathogen-drug combinations in 204 countries (1). According to a global survey conducted in 2019, it was revealed that antimicrobial resistance claimed more lives than HIV/AIDS or malaria (2). Within bacterial cells, multidrug resistance (MDR) manifests in two distinct forms: either through the accumulation of diverse drug-resistant components in multiple genes or by upregulating genetic expression for multidrug efflux pumps (3, 4).

Tackling this challenge is daunting. The heart of the problem lies in the intricacies of multidrug therapy. The absence of chemical bonds between different drug molecules means that when administered, these molecules reach individual bacteria separately. This isolated delivery inadvertently enhances resistance against each drug, amplifying the complexities of conventional multidrug therapy. Consequently, eradicating multidrug-resistant bacteria becomes an uphill battle, prolonging treatment times and inadvertently promoting further resistance. As a result, the extended duration of treatment carries an insidious risk: the potential escalation of multidrug-resistant infections within society. This, in turn, heightens the possibility of a widespread epidemic. Addressing these critical issues is paramount in our quest to confront the escalating threat of antibiotic resistance.

In the battle against multidrug-resistant bacteria, this hypothesis introduces an unprecedented novel approach, aiming to deliver all the drug molecules to every targeted bacteria cell in a conjugative manner. The cocrystals (5, 6) are formed by exploiting supramolecular synthons (7–9) to form the bond between two or more molecules and crystallizing them together. The use of coformer with a drug molecule while crystallization generally helps promote permeability, solubility, and thermal stability, while reducing brittleness (5, 10). Various supramolecular bonds such as π-π stacking (11), halogen bonding (12–14), hydrogen bonding (15, 16), ion interactions (17), and van der Waals’ interactions (18) can facilitate cocrystal formation. By creating cocrystals of different drugs tailored (19, 20) for the same type of bacteria, it becomes possible to administer all drug molecules simultaneously to a single bacterium. This simultaneous attack by multiple drug molecules on a single bacterium can be more effective than the traditional multidrug therapy protocol. Cocrystal formation (19) enhances the solubility and flexibility of drug molecules (19), negating the requirement for pharmaceutical excipients (19). Pharmaceutical excipients are essential for safely delivering medication molecules to specific organs. By combining essential pharmacological molecules with their complementary counterparts, we can enhance plasticity, solubility, and tabletability (21). This innovative approach could entirely replace the need for excipients, particularly in cases of multidrug cocrystallization (20). By adopting this strategy, we aim to enhance patient outcomes by bolstering the efficacy of medications against bacterial infections.

**Scheme 1:**
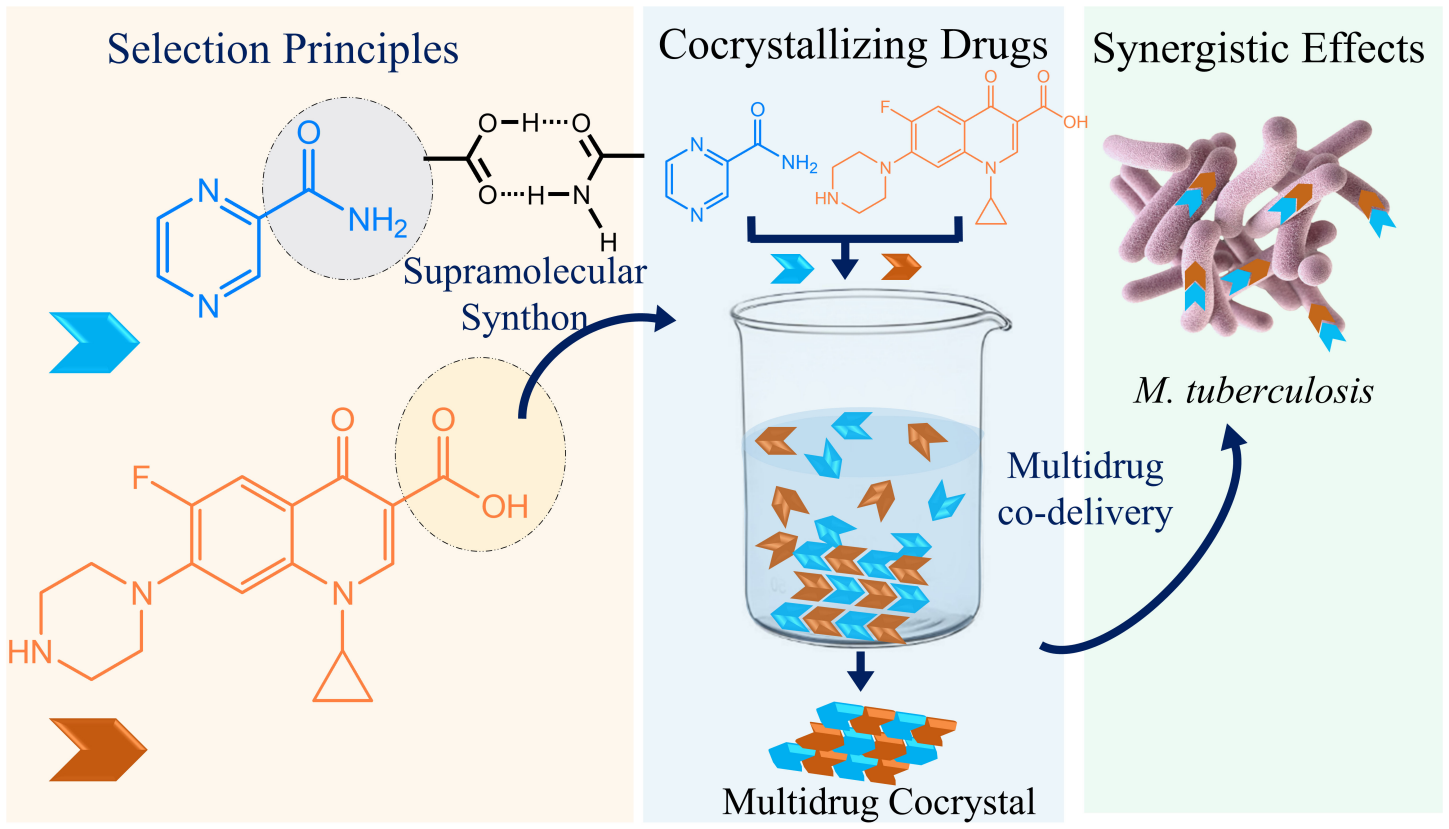
A schematic presentation depicts how multidrug therapy can be improved. The complementary tuberculosis drugs with the potentiality of forming carboxylic acid–carboxamide synthon may form cocrystals in certain solutions and possibly can be codelivered to treat tuberculosis.

The formation of these cocrystals involves employing techniques like slow evaporation, hydrothermal procedures, and solvent diffusion (19) in different solvent mediums, ensuring the necessary conditions for cocrystal development (**Scheme 1**). Notably, six major pathogens, including *Escherichia coli*, *Staphylococcus aureus*, *Klebsiella pneumoniae*, *Streptococcus pneumoniae*, *Acinetobacter baumannii*, and *Pseudomonas aeruginosa*, were collectively responsible for approximately 929,000 deaths in 2019, underscoring the urgency of innovative solutions in combatting multidrug-resistant bacteria (1).

## 2 Multidrug resistant bacteria

The development of drug-resistant bacteria poses a significant challenge in their eradication. Here, a selection of bacteria is used to demonstrate the effectiveness of cocrystal therapy in combating them as well. The Gram-negative pathogens have raised various levels of drug resistance like multidrug resistance (MDR), pan drug resistance (PDR), and extensive drug resistance (XDR) (22), making them highly lethal in epidemic scenarios. To establish standardized terminology, the Centers for Disease Control and Prevention (CDC) and the European Centre for Disease Prevention and Control (ECDC) collaborated to provide clear definitions. XDR is characterized by non-susceptibility to one or more antimicrobial classes out of the two available. MDR occurs when drug resistance is observed in at least one agent from three or more antimicrobial groups. PDR is defined when drug resistance is present across all antimicrobial classes, leaving no agents vulnerable to the organism.

Infections caused by Gram-negative bacteria such as *Acinetobacter baumannii, Escherichia coli*, *Pseudomonas aeruginosa*, and *Klebsiella pneumonia* have alarmingly high mortality rates. The extensive and prolonged treatments, along with meticulous medical care and infection prevention measures, result in substantial financial expenditure. Providing optimal care for critical patients involves understanding the geographical patterns of resistance and individual factors contributing to resistance. Despite global initiatives to discover new treatments for MDR Gram-negative infections, progress has been limited. Addressing the challenges posed by MDR requires exploring novel combinations of existing antibiotics, β-lactamase inhibitors like relebactam, avibactam, or vaborbactam are often combined with conventional antibiotics such as cephalosporins or carbapenems to enhance their effectiveness against targeted bacteria (23).

### 2.1 Acinetobacter baumannii

*Acinetobacter baumannii* is another gram-negative nonfermenting genus, that lives in diverse environments such as soil, water, vegetables, various animals, and human hosts. It is an almost round, rod-shaped bacteria, that resides in the flora of mucous membranes and human skin. Both community and hospital-acquired infections are primarily attributed to *A. calcoaceticus-A. baumannii* complex (24). Within the United States, *A. baumannii* instigates approximately 12,000 healthcare-associated infections annually, with 7,200 cases evolving into multidrug-resistant strains, leading to 500 fatalities (25). Equipment within hospitals, such as mechanical ventilation systems, dialysis machines, and water sources, frequently harbours *A. baumannii*, despite its presence in the mucous membranes and skin of healthcare professionals or patients (26). Previously deemed an opportunistic organism with minimal pathogenicity, Acinetobacter was perceived as having low impact. However, the World Health Organization (WHO) has recently categorized it as a top-priority pathogen, emphasizing the urgent need for antibiotic development targeting this bacterium (27). *A. baumannii* is considered an extremely life-threatening pathogen. This bacterium commonly exhibits substantial resistance to antimicrobial agents. Multidrug-resistant strains of *A. baumannii* frequently exacerbate patient conditions due to inadequate initial therapy, limited treatment alternatives, and the heightened toxicity of available treatments. Drug resistance, either acquired or intrinsic, is facilitated by various factors, such as alterations in membrane permeability or the production of beta-lactamases, which degrade beta-lactam antibiotics.

Among gram-negative bacilli, beta-lactamases stand as the foremost contributor to bacterial resistance (28). Carbapenem resistance stems from changes in penicillin-binding proteins and the action of efflux pumps. Newer medications like eravacycline, cefiderocol, and ETX2514, alongside established ones such as aminoglycosides (29), polymyxins E and B, piperacillin/tazobactam, carbapenems, sulbactam, tigecycline, and could be co-administered to create complementary drug-based cocrystals as a potential strategy.

### 2.2 Pseudomonas aeruginosa

*Pseudomonas aeruginosa*, a rod-shaped multidrug-resistant Gram-negative aerobic–facultatively anaerobic, bacterium prevalent in soil, water, and healthcare settings, poses a significant threat as a source of nosocomial infections. Through the denitrification enzyme, *P. aeruginosa* reduces nitrate to molecular nitrogen under anaerobic respiration. It targets various systems, including the bloodstream, urinary tract, and ventilators (leading to pneumonia) (30). Its adaptability in acquiring new genetic elements enables resistance to multiple antimicrobials. The bacterium’s cell wall’s low permeability hampers antibiotic uptake, contributing to its resistance profile. Typically, it targets individuals with weakened immune systems; however, it can also infect those with a fully functional immune system, as seen in cases like hot tub folliculitis.

Carbapenems, initially the primary line of defense, face challenges due to mechanisms such as efflux pumps, loss of porin, and reduced drug permeability, leading to resistance (31). Carbapenem-resistant *P. aeruginosa* (PARC) has emerged as a critical concern, contributing to hospital outbreaks in multiple countries (32). To address this, novel drugs like Cefiderocol, Ceftolozane-tazobactam, and Ceftazidime-avibactam are being utilized against Pseudomonas species (33). The genome structure of *Pseudomonas aeruginosa* comprises a notably large circular chromosome (ranging from 5.5 to 6.8 Mb) housing between 5,500 and 6,000 open reading frames. Additionally, depending on the strain, it may contain plasmids of varying sizes (34). A comparison of 389 genomes from diverse *P. aeruginosa* strains revealed that only 17.5% of the genome is commonly shared, constituting the *P. aeruginosa* core genome (35).

### 2.3 Staphylococcus aureus

*Staphylococcus aureus*, a resilient gram-positive bacterium resistant to multiple drugs, lives in diverse environments such as soil, air, and water, and colonizes at skin and human nose. It causes severe illnesses like pneumonia, septicemia, endocarditis, meningitis, and systemic infections, posing a considerable risk of mortality (36). Initially, *S. aureus* infections responded well to penicillin until the bacterium swiftly acquired beta-lactamase through plasmid-encoded instructions, rendering penicillin ineffective in clinical settings.

To tackle this challenge, methicillin, a semisynthetic beta-lactamase-resistant antibiotic, was developed and employed in 1959 against penicillin-resistant strains. However, methicillin resistance emerged rapidly (37). By 1961, methicillin-resistant *S. aureus* had triggered a global outbreak, ranking among the top three threatening infectious diseases for human health.

In 2011, the Centers for Disease Control and Prevention reported over 80,000 illnesses and 11,000 fatalities attributed to Methicillin-resistant *S. aureus* (MRSA) (38). Initially confined within hospital boundaries, MRSA transitioned to community settings by the 1990s. Certain strains have been identified in pet animals such as cattle, chickens, pigs and horses, leading to conditions like pneumonia, necrotizing fasciitis and endocarditis since 1975 (39, 40). Presently, daptomycin and Vancomycin stand as common antibiotics used against *S. aureus* infections (41), but methicillin-resistant strains are beginning to display an MDan phenotype, exhibiting resistance or susceptibility to such antibiotics. Certain penicillin-derived narrow-spectrum beta-lactam antibiotics—such as cloxacillin, flucloxacillin, dicloxacillin, Methicillin, nafcillin and oxacillin, —hold potential for treating *S. aureus* infections and may be utilized in cocrystallized forms to address multidrug-resistant bacterial strains.

### 2.4 Mycobacterium tuberculosis

Following the aftermath of the COVID-19 pandemic, tuberculosis (TB) has emerged as a looming threat due to the scarcity of medicines in remote regions. The focus on immediate COVID-19 treatment led to the neglect of other chronic diseases such as TB and malignancies. As a result, TB patients inconsistently received their medications, leading to the emergence of multidrug resistance in the TB-causing bacteria *Mycobacterium tuberculosis*. It is noteworthy that TB has claimed more lives than any other infectious disease over the past 2000 years. Instead of continuously synthesizing new TB drugs, a more efficient approach lies in preparing cocrystals of existing drug molecules. This approach minimizes scientific efforts and time. In addition to this primary threat, other major multidrug-resistant bacteria are also raising concerns regarding the certainty of life (21). Individuals infected with HIV are particularly vulnerable to mycobacterial diseases (42). By gaining a comprehensive understanding of the *defense mechanisms of M. Tuberculosis* and countering them with complementary drug molecules, researchers can identify suitable drugs for the development of cocrystals. *M. tuberculosis*, classified as weakly Gram-positive (43), is a pathogenic bacterium prevalent in a series of animals, including humans, goats, cats, dogs, rabbits, pigs, deer, and badgers (42). Identification methods involve acid-fast stains like Ziehl-Neelsen or fluorescent stains such as auramine (44). This bacterium has the capacity to infect diverse body regions, like the spine, brain, and kidneys. It’s noteworthy that in certain individuals, *M. tuberculosis* can remain asymptomatic, leading to the condition termed latent TB. In the 2022 Global Tuberculosis Report released by the World Health Organization (WHO) (45), findings revealed 450,000 fresh instances of RR-TB worldwide in 2021, with China accounting for 33,000 new cases of MDR-TB/RR-TB.

**Figure 1:**
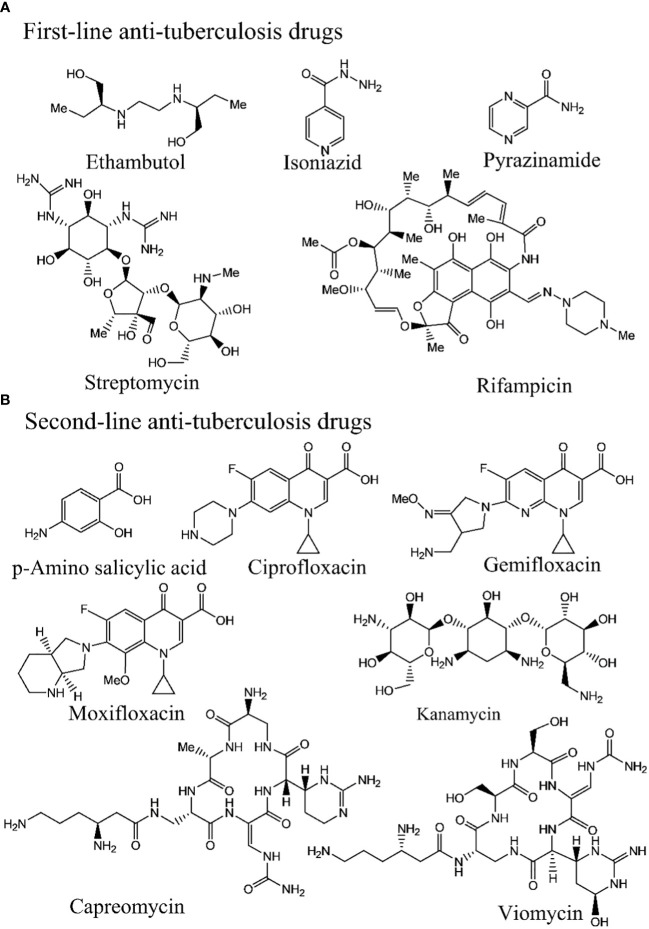
Various first- and second-line tuberculosis drugs are presented.

Tuberculosis (TB) medications are broadly categorized into first-line drugs (**Figure 1A**), (eg. rifampicin, isoniazid, ethambutol, pyrazinamide, and streptomycin, second-line drugs (**Figure 1B**), (amikacin bedaquiline, clofazimine, cycloserine, linezolid, moxifloxacin, levofloxacin, para-aminosalicylic acid, and propylthiouracil (46). The treatment duration for rifampicin- resistant TB (RR-TB) typically spans 18–20 months (46), comprising a 6-month intensive phase followed by a 12–14-month continuation phase. Tailoring the treatment strategy to the patient’s drug resistance status is customary (47, 48). The first- and second-line tuberculosis drug molecules are presented in **Figure 1**. As treatment for susceptible TB has remained largely unchanged for the past four decades and with the emergence of drug resistance, there is an urgent need for new drugs and treatment regimens. A tremendous effort is going on in developing the new drugs or regimens for the TB treatment (21, 49–55).

## 3 Multidrug resistive mechanism

It becomes important to know the multidrug resistance mechanism of every different bacterium to design both molecular drugs or complementary supramolecular drug associates. The molecular drug has to be quite new to the bacteria so that the unexperienced bacteria will be clueless and can be destroyed consequently. However, in the case of a supramolecular drug associate, two associated drugs should complementarily work to destroy the bacterial defense mechanism. Bacterial multidrug resistivity works under a few certain pathways (56). In the 1^st^ case, the bacteria obtain numerous drug-resistant genes via the horizontal plasmid transferring through the bacterial conjugation. The 2^nd^ category works by increasing the genetic expression, coded with multidrug efflux pumps. However, the organisms have evolved increasingly complex mechanisms of resistance (57).

### 3.1 Transferring plasmid through horizontal conjugation

**Figure 2:**
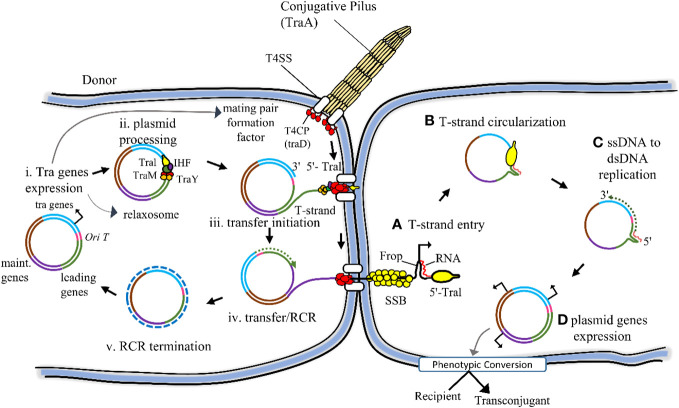
Schematically conjugational transfer of the F plasmid from the donor to the recipient cell is represented here. The backbone of the F plasmid consists of various components: the tra regions, encompassing all genes responsible for conjugational transfer (depicted in light blue); the origin of transfer (oriT) highlighted in red; the leading region (depicted in green), which is the initial segment transferred into the recipient cell; and the maintenance region (depicted in dark blue), playing a role in plasmid replication and partition. (i) The conjugation gets started by the expression of the tra gene. Certain Tra proteins are responsible for assembling both the T4SS and the conjugative pilus. These structures play a pivotal role in attracting recipient cells and facilitating the stabilization of mating pairs during the process of conjugation. (ii) Additional Tra proteins, namely TraI, TraM, and TraY, make up the relaxosome complex. Working in tandem with the integration host factor (IHF), they specifically bind to the oriT site on the plasmid. Their role is crucial in preparing the plasmid for transfer by initiating the nicking reaction through the TraI relaxase enzyme. (iii) The transfer of the T-strand through the T4SS is triggered by the interaction between the Type IV Coupling Protein (T4CP) and Relaxosome. This interaction marks the initiation point for the transfer process. (iv, v) As the TraI-bound T-strand moves to the recipient, the donor undergoes Rolling Circle Replication (RCR), converting single-stranded DNA (ssDNA) into double-stranded DNA (dsDNA) concurrently. (A) Upon entering the recipient, the single-stranded DNA (ssDNA) T-strand becomes enveloped by the host chromosomal SSB. Simultaneously, the single-stranded promoter Frpo takes on a stem-loop structure that the host RNA polymerase identifies to kickstart RNA primer synthesis. (B) TraI facilitates the circularization process of the completely internalized T-strand. (C) The host DNA polymerase identifies the RNA-DNA duplex, triggering the initiation of the complementary strand synthesis. (D) Upon completion of the conversion from ssDNA to dsDNA within the plasmid, the expression of plasmid genes triggers a phenotypic transformation in the recipient cell, turning it into a transconjugant cell. (Ref 3).

The host-plasmid pairs promote multidrug resistance in both Gram-negative and Gram-positive bacteria (58). MDR bacteria disseminate their drug resistance to neighboring bacteria via the horizontal transfer of plasmids, which harbour antibiotic-resistance genes. The stabilizing factors within plasmids during bacterial conjugation further enhance multidrug resistance, as bacteria acquire not only their plasmids but also novel drug-resistant plasmids (**Figure 2**) (59). After removing the antibiotics, the emergence of MDR is facilitated by a crucial stabilizing factor: the coevolution of host-plasmid pairs under antibiotic selection. This process involves the exchange of two distinct plasmids from different bacteria (*Escherichia coli* and *Klebsiella pneumonia*). In this scenario, evolution favoured the increased stability of a plasmid.

### 3.2 Efflux pumps

**Figure 3:**
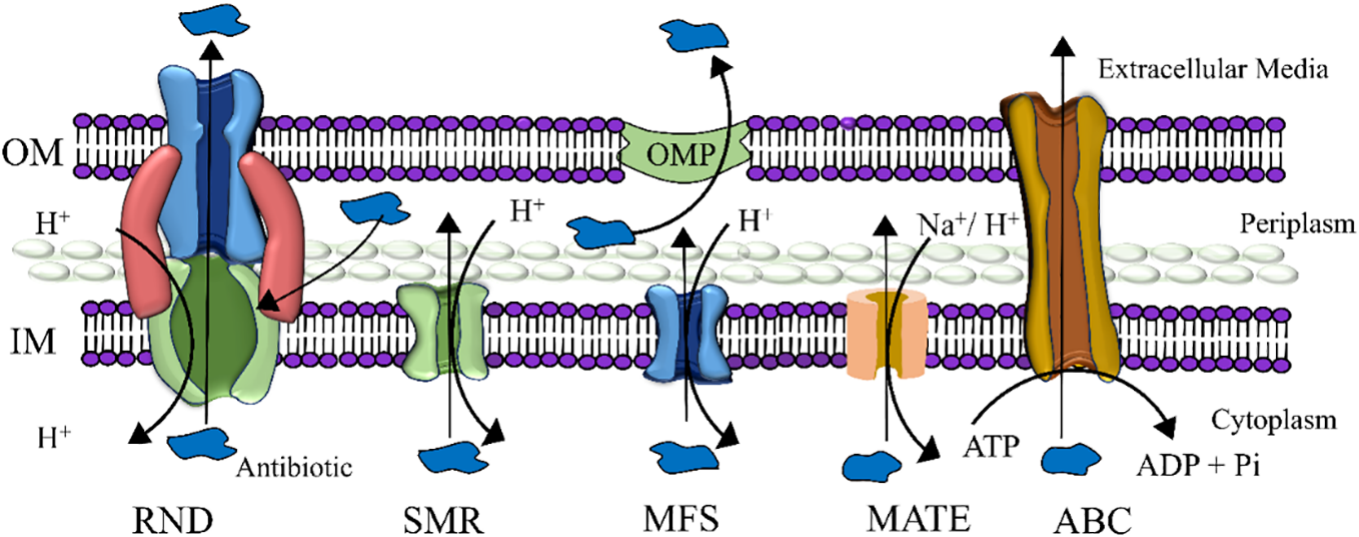
Five major families of efflux pumps are schematically presented here. (i) resistance-nodulation-division (RND), (ii) small multidrug resistance (SMR), (iii) major facilitator superfamily (MFS), (iv) multidrug and toxic compound extrusion (MATE), and (v) ATP-binding cassette (ABC) superfamily. The abbreviations used in this context expand as follows: OMP, Outer membrane protein; OM, Outer membrane; ATP, Adenosine triphosphate; ADP, Adenosine diphosphate; IM, Inner membrane.

**Figure 4:**
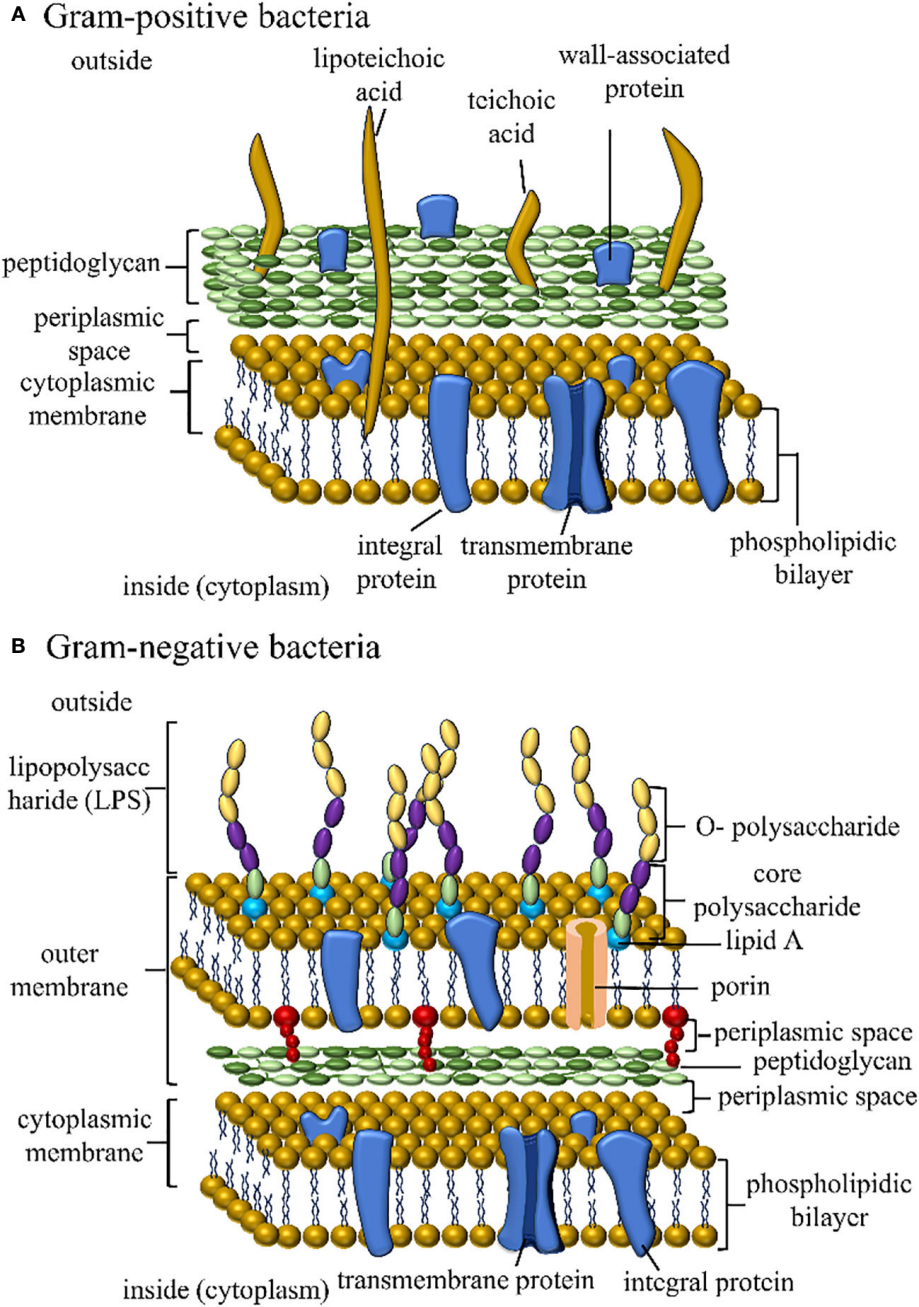
The figure illustrates the cell membrane structures of Gram-positive and Gram-negative bacteria.

The cell membrane that houses efflux pumps (**Figure 3**) (56) is the inner membrane transporter, which not only expels antibiotics (60) but also discharges various harmful substances, such as pollutants, heavy metals, and antimicrobial agents produced by competing organisms (61). Bacteria employ antibiotic efflux as a key defense mechanism, expelling antibiotics from their cellular interior into the external environment through specialized transporter proteins known as efflux pumps. Given the decline in novel antibiotic discovery, targeting these pumps has emerged as an appealing strategy. Efflux pump inhibitors (EPIs) are molecules capable of blocking these pumps, offering promise as therapeutic agents to reinvigorate the effectiveness of antibiotics that have lost their potency against bacterial pathogens. These inhibitors operate via diverse mechanisms, sourced from both natural and synthetic origins. **Figure 4** schematically represents the compares the cell membranes of both Gram-positive (**Figure 4A**) and Gram-negative (**Figure 4B**) bacteria. Multidrug efflux pumps play a pivotal role in conferring resistance among bacterial pathogens, encompassing acquired, intrinsic, and phenotypic traits. Their expression is usually tightly regulated, offering the possibility of achieving heightened and temporary levels of expression via specific biological triggers or effectors.

### 3.3 Bacterial enzymes

Another significant drug-resistant mechanism prevalent in multidrug-resistant strains involves bacterial enzymes (62). Based on the drug-resistive mechanism; the enzymes can be classified can be classified further (62). The bacteria can i) modify their enzymes to target the antibiotic agents (63); ii) improve the enzymes to target intracellular sites; iii) exploit the cellular metabolic reactions and iii) carry out the enzymatic transformation of antibiotics. All the antibiotic-resistant genes are denoted as resistome (64). Gene-encoded enzymes on the chromosome shield pathogens, safeguarding antibiotics from modifying their intended targets (62).

### 3.4 Losing porins

Certain antibiotics and drugs can be readily administered to Gram-positive bacteria due to their lack of an outer membrane (65). While the outer membrane is resistant to many antibiotics, the presence of porins in Gram-negative bacteria makes this membrane permeable, allowing easy access to essential needs such as nutrition, water, food, or ions. Antibiotics can also come through this way. The Gram-negative bacteria enhance their resistance to antibiotics by losing porins, hindering the entry of antibiotics into the cell and strengthening the bacteria’s defense against them.

**Figure 5:**
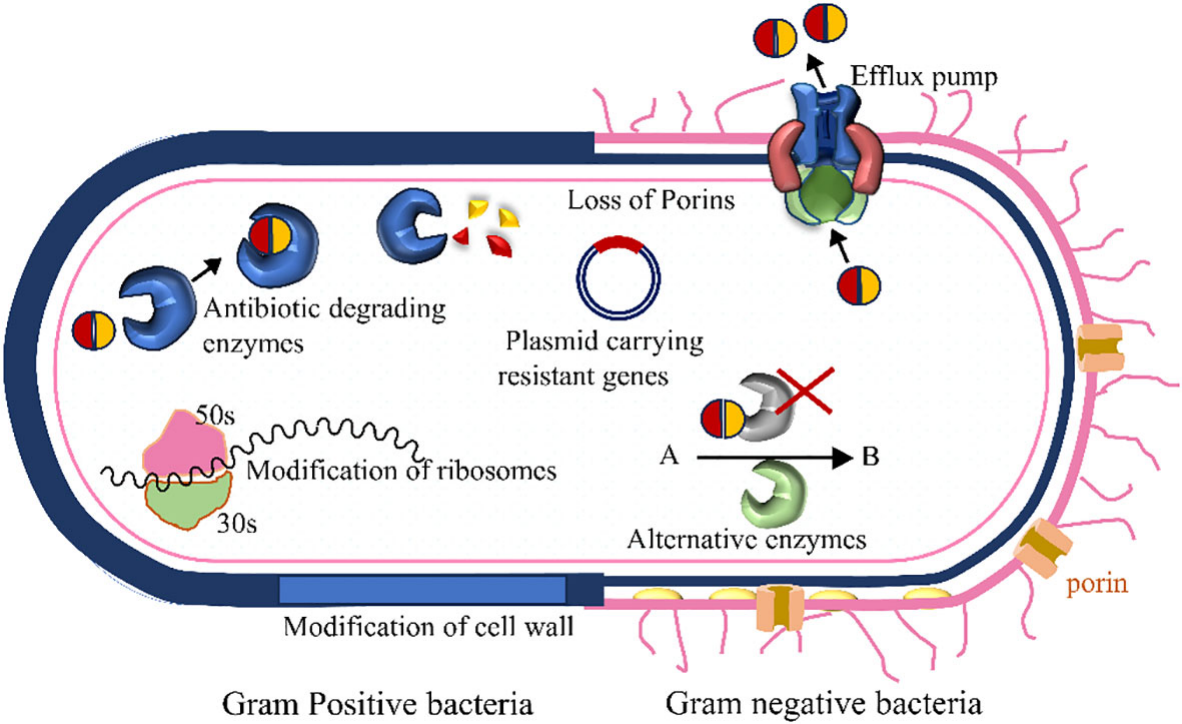
Several antibiotic resistance mechanisms in bacteria are presented here schematically. The left and right sides denote the presentation of Gram-positive and Gram-negative bacteria, respectively. Bacteria acquire enzymes that neutralize the drugs, efflux pumps actively move particular or multiple antibiotics out of the cell, alternative metabolic routes replace those blocked by the medication, the antibiotic’s target site undergoes modification, reducing the drug’s affinity to the binding sites, and decreased permeability results in reduced drug accumulation within the cell.

Besides these major antibiotic resistance mechanisms, the bacteria can raise the drug resistance through some other alternative process as well (**Figure 5**). For example, it can modify cell walls and ribosomes.

## 4 The limitations and challenges of present therapies

The rise of resistance to antimicrobial agents has become a significant contributor to global morbidity and mortality. In the early 1900s, the introduction of antibiotics initially appeared to signal victory against microorganisms. However, it became evident that these microorganisms could develop resistance to the drugs being utilized. As a result, scientists across various fields have been striving to create new drugs or treatment regimens, emphasizing multidrug therapy. Yet, observing the challenges faced, it’s crucial to introspect the current treatment approaches and refine the philosophy driving multidrug therapy’s advancement.

### 4.1 Limitations of multidrug therapy

Multidrug-resistant bacteria enhance their resistance by exchanging multiple genes through conjugation between bacterial cells or by utilizing multidrug efflux pumps. Consequently, these bacteria can develop resistance against previously unknown drugs within a few years through these mechanisms. To counter this challenge, a combination of multiple drugs can be administered concurrently. In this approach, a combination of multiple drugs is administered simultaneously. Each drug acts independently, targeting the bacteria one after another until the bacterium is effectively eliminated. Out of the philosophy of using multiple drugs, one very common multidrug-resistant bacterium-killing regimen is multidrug therapy. Though at present it is one of the most convincing ways to fight against drug-resistant bacteria, the following drawbacks urge the scientific society to surge the new regimen (66).

#### 4.1.1 Development of resistance

As multiple drug molecules do not have their mutual bonds, they reach the bacteria cells one by one. In this process, a bacterium often gets enough time to gain drug resistance (21). It enhances the treatment time and raises the risk of spreading multidrug-resistant bacteria in society. As the strains acquire drug resistance, they gradually transition from MDR or XDR to PDR, making it increasingly challenging over time to identify the remaining effective drugs.

#### 4.1.2 Toxicity

Using multiple drugs simultaneously can increase the risk of adverse side effects and toxicity in patients. Some drugs may have overlapping toxic effects, leading to complications and limiting the dosage that can be administered safely. Oral, abbreviated multi-drug resistant tuberculosis (MDR-TB) treatments have the potential to lessen the inconvenience, discomfort, and side effects associated with these regimens. This could enhance adherence rates and improve the chances of successful completion of the treatment (67).

#### 4.1.3 Complexity of administration

Coordinating the administration of multiple drugs, each with specific dosages and schedules, can be challenging. This complexity can lead to errors in medication adherence, reducing the overall effectiveness of the therapy. Medication errors include inappropriate medication, wrong dose, drug-drug interaction, allergic reaction, incorrect delivery path, lack of proper education in patients etc. Annually, in the United States, approximately 7,000 to 9,000 individuals lose their lives due to medication errors (68).

#### 4.1.4 Cost

Procuring multiple drugs and conducting extensive testing to determine the most effective combination can be financially burdensome. This cost factor can limit the accessibility of multidrug therapy, especially in resource-limited healthcare settings. We may have to spend $16.7 trillion by 2050 for the Multidrug-resistant TB only (69).

#### 4.1.5 Potential drug-drug interaction

When multiple drugs are used simultaneously, there is a risk of drug interactions. These interactions can alter the effectiveness of the drugs or exacerbate side effects, complicating the treatment process (70).

The use of multiple drugs can exert selective pressure on bacteria, favouring the survival and proliferation of resistant strains. This phenomenon can further exacerbate the problem of antibiotic resistance in the long term.

### 4.2 Limitations of universal drug regimen

One seminal paper on the short-course regimens for treating tuberculosis raised the hope of discovering the universal drug regimen in 1986 (71). Regrettably, the *Mycobacterium tuberculosis* strains developed rifampicin resistivity and the treatment policy became ineffective for a significant number of patients (72). The WHO expressed deep concern over the fact that in 2016, over 600,000 individuals were affected by rifampicin-resistant tuberculosis. According to their recent report, 17,000 mutant varieties of tuberculosis have been published, along with their corresponding drug resistance profiles (73). The identification of the variants and selecting their corresponding drug regimen is a daunting task. It results in the consumption of unwanted drugs and generates side effects. To address this issue, we need to find a universal drug regimen, by which every drug-resistant bacterium can be addressed. Inspired by this hypothesis, universal drug regimen preparation has been on the way since the last decade (74). Identifying a handful of complementary drugs for universal drug therapy is a topic of debate, but once the universal drug regimen is developed, all TB patients can be treated in the same way and the patients can be treated in a general way without ‘personalized medicinal’ selection according to the *M. tuberculosis* variant in the body. However, developing a perfect combination of several novel drugs in preparing a universal drug regimen still fails to address a series of medical concerns even if it comes to society. The determination of potency, unknown interactions between drugs, excessive medication side effects, and the overall cost of treatment continue to pose challenges similar to the multidrug treatment protocol.

Administering drug molecules individually to bacteria may gradually increase drug resistance. Attaining universal therapy goes beyond simply combining complementary drug molecules; it necessitates the development of cocrystals involving these complementary candidates to ensure the effective delivery of drug molecules to the bacterial cells.

### 4.3 AI for designing multidrug-resistant drug

Like other sectors (75–77), the drug molecules, including the antibiotic are also designed with the help of artificial intelligence (78). *Acinetobacter baumannii*, a resilient Gram-negative pathogen commonly found in healthcare settings, often resists multiple drugs, making it difficult to find effective antibiotics through traditional methods. However, leveraging machine learning has significantly accelerated the exploration of chemical space, increasing the chances of discovering new compounds to combat this bacterium. In a recent study, Liu et al. screened around 7,500 molecules to identify those capable of inhibiting *A. baumannii* growth *in vitro* and discovered the antibiotic abaucin. Using a neural network trained on this dataset, they predicted the efficacy of structurally unique compounds against *A. baumannii*, leading to the discovery of abaucin—an antibacterial agent demonstrating a specific impact on *A. baumannii* (79).

In a similar study, Talat et al. (80) employed AI in uncovering various beta-lactamase inhibitors and alternative antibiotics derived from antimicrobial peptides (AMPs), nonribosomal peptides, bacteriocins, and marine natural products. With the expansion of next-generation sequencing, the volume of data obtained is skyrocketing, making manual extraction of low-risk antimicrobial resistance drugs from such vast datasets nearly impossible. Raban et al. illustrate AI’s exceptional efficacy in developing antibiotics, serving as a robust response to the concerning rise in antimicrobial resistance rates (81). The AI system is also efficient in predicting the drug delivery mechanism so that the specific drug can be developed against certain drug-resistant strains (82). This system includes drug development, can predict drug resistance, select the proper drug combination, optimize the drug dose, and also can improve the drug delivery system. The prospect of machine learning methods (a branch of AI) in forecasting whether a patient might develop an MDR pathogen within the initial 48 hours of ICU admission holds significant promise (83).

Theoretically finding an appropriate molecule by AI becomes a normal tusk and then the movement can further be accelerated by exploiting the robot scientist. Experimentally a robot scientist can work for 24 hours multiplied by 365 days. In finding the appropriate water oxidation catalyst experimentally, an autonomous robot spanned eight days, conducting 688 experiments within a complex experimental framework involving ten variable places. These actions were steered by a batched Bayesian search algorithm (84–86). The autonomous search process pinpointed photocatalyst mixtures that exhibited activity six times greater than the initial formulations. It effectively identified advantageous components while excluding detrimental ones. The robot is also capable of optimizing organic reaction conditions to improve yields and simplify the process (87). A different group (88) develops a free-roaming (89–92) dexterous (93, 94) robot, that automates the role of the researcher, rather than directly manipulating the instruments. Such robot scientists can also be used for developing molecular drugs. Now, once the drugs are designed by AI and can be experimentally prepared by the robot scientists, can be forwarded for medical trials within a week so that the quick harmonization can bring them to the market quickly.

Furthermore, the last decade initiated the multistep flow synthesis (95–97) so that multistep organic reactions can be carried out autonomously and a whole system can run fast in finding effective antibacterial drugs. So, in future, getting new antibacterial materials may not be that difficult, but inappropriate use can always develop drug resistivity. Additionally, employing numerous or diverse arrays of drugs can foster a broad spectrum of drug resistance in pathogens. This could significantly complicate the search for suitable drugs in the context of multidrug therapy in the coming years. Therefore, the supramolecular assembly formation or cocrystallization of complementary multidrug can stabilize rapid pharmaceutical activities, reducing costs and streamlining the drug selection process associated with this extensive effort. For multidrug administration to be effective, a synergistic action is crucial, necessitating the simultaneous delivery of multiple drugs to a bacterial cell. Whether using conventional multidrug therapy or a universal drug regimen, the current treatment protocol involves drugs reaching the bacteria separately, providing them an opportunity to develop resistance. Loading multiple drugs into a nanocontainer or carrier isn’t feasible with present technology, and it doesn’t facilitate the simultaneous delivery of drugs to the bacteria. To address all these issues, the formation of supramolecular drug associations is essential to effectively implement the philosophy of multidrug therapy.

### 4.4 Dendrimers in codrug delivery

In the realm of codrug delivery, the impact of supramolecular bonding, ranging from weak to robust, plays a pivotal role in determining the availability of drug associates to combat bacteria. This has prompted a shift towards incorporating dendrimers as key players in the delivery of multidrugs. Dendrimers, belonging to a distinctive category of macromolecules, seamlessly blend the structural attributes of individual molecules with the extended characteristics of polymers, making them a promising avenue for advancing codrug delivery strategies. Their three-dimensional structure, combined with a diverse range of additional substrates available for assembly, provides multifaceted potential for applications in medicine, diagnostics, and environmental domains. Particularly noteworthy are peptide dendrimers, acting as conduits for transporting therapeutic substances like synthetic vaccines targeting parasites, bacteria, and viruses, contrast agents employed in MRI, antibodies, and genetic material (98). Dendrimers, possessing highly branched structures and easily modifiable surfaces, hold significant promise for functionalization and conjugation with drugs and DNA/RNA. Their precisely synthesizable controlled architecture enables fine-tuning of characteristics such as charge, shape, solubility, and size in carrying wide ranges of drugs and gens (99, 100).

Most importantly, Siriwardena et al. developed one second-generation (G2) peptide dendrimers equipped with a fatty acid chain at the dendrimer core, demonstrating their effectiveness in eradicating Gram-negative MDR bacteria, like *Pseudomonas aeruginosa*, *Acinetobacter baumannii*. Another dendrimer, namely TNS18, can exhibit activity against the Gram-positive *Staphylococcus aureus*, which was methicillin-resistant in nature. It also shows the antibiotic activities against *A. baumannii* and *E. coli* (101).

The dendrimers can be used as the drug carrier against the tuberculosis bacteria (102, 103). These dendrimers can encapsulate several antibiotics. If we select the dendrimers with their selective antibacterial activities with the complementary drugs, then besides forming cocrystals, we can administer the multidrug.

There remain several challenges in utilizing dendrimers for co-delivering multiple drugs to specific bacterial cells. The cytotoxic impact of dendrimers is intricately tied to the properties of their surface groups, particularly their charge. Regardless of the surface composition and molecular structure, cationic dendrimers exhibit heightened cytotoxicity and hemolytic tendencies compared to their anionic and neutral counterparts (104, 105). Branching in dendrimers also enhances the cytotoxicity. This elevated toxicity is primarily due to their non-selective attraction to cell membranes that possess a negative charge. Notably, this cytotoxic effect varies with the dendrimer generation and is amplified by specific surface groups (106), while *in vitro* studies reveal that neutral and negatively charged dendrimers do not induce cytotoxic effects. Mitigating the toxicity of positively charged dendrimers is pivotal to their application in diagnostics and therapeutics. Consequently, strategies involving chemical modifications of terminal groups have been proposed. These alterations aim to enhance targeting capabilities, substantially extend the duration of the dendrimers in the bloodstream, and refine their design to achieve better distribution within the body and enhanced biocompatibility (107). Finally, the dendrimers cannot guarantee the simultaneous delivery of all encapsulated drug components to a bacterial cell, potentially contributing to drug resistance in bacteria.

### 4.5 Challenges of developing a new ideal therapy

Multidrug-resistant bacteria heighten their resistance in the presence of external drug molecules or foreign bodies. Multiple drugs are administered in hopes that at least one will effectively kill the bacteria before it becomes entirely resistant to the treatment. While this approach seems logical, the challenge lies in the protocol for drug administration. In multidrug therapy, simultaneous delivery of multiple drugs to a bacterial cell is never guaranteed. This situation raises drug resistance and facilitates the sharing of genetic information through plasmid transfer among nearby bacteria, culminating in the development of resistant bacterial colonies and subsequent treatment failures.

## 5 Present complementary multidrug regimens

We’ve observed that combined binary antibiotic systems present in the multidrug regimens work efficiently in killing MDR bacteria. Among the two antibiotic molecules, one is prone to be attacked by the bacterial defence mechanism. Bacteria destroy the molecule by attacking a class of specific functional backbones. For example, bacterial beta-lactamase enzyme destroys the lactam-containing antibiotic molecules. In this situation, if a 2nd antibiotic molecule prevents beta-lactamase formation or production, then the lactam-containing antibiotic can be safeguarded from the bacterial defence mechanism and kill the bacteria. Through this preventing mechanism, a binary set of antibiotics can destroy the bacteria cells.

### 5.1 Ceftazidime/avibactam

Ceftazidime is a third-generation β-lactam antibacterial drug useful for the treatment of a series of gram-positive (like *Staphylococcus aureus*, *Streptococcus pneumoniae*, *Streptococcus pyogenes*, *Streptococcus agalactiae*) and gram-negative bacteria (like *Citrobacter* species, *Enterobacter* species, *Escherichia coli, Klebsiella* species, *Haemophilus influenzae*, *Pseudomonas aeruginosa* etc). It’s employed in the treatment of infections affecting the lower respiratory tractbloodstream, joints, skin, urinary tract, abdomen, and meningitis. Avibactam, a novel β-lactamase inhibitor akin to diazabicyclooctanes (DBO), reversibly binds to β-lactamase enzymes, facilitating their recycling and subsequent binding to broad-spectrum β-lactamases. This mechanism significantly amplifies the activity of ceftazidime by more than a thousand-fold. Avibactam demonstrates efficacy in managing pyelonephritis, intricate intra-abdominal infections (cIAI) (23), and intricate urinary tract infections (cUTI). It offers broad coverage against Ambler Classes A, C, and D pathogens (108, 109). The FDA greenlit Ceftazidime/avibactam’s usage in the USA back in 2015 for managing complicated intra-abdominal infections (when used alongside metronidazole) and complicated urinary tract infections, encompassing pyelonephritis, specifically for patients aged 18 years and older (57, 110).

Avibactam shields ceftazidime from hydrolysis by several bacterial β-lactamase enzymes, encompassing Klebsiella pneumoniae carbapenemase (KPC), extended-spectrum β-lactamase (ESBL), class C (AmpC), and several class D β-lactamases. However, in the presence of metallo-β-lactamases like New Delhi metallo-β-lactamase (NDM), Verona integron-encoded metallo-β-lactamase (VIM), and imipenemase (IMP), avibactam loses its protective effect on ceftazidime against hydrolysis (111).

During a research investigation evaluating antimicrobial effectiveness against carbapenem-resistant Enterobacterales found in ICUs across Taiwan, ceftazidime/avibactam showcased high susceptibility rates: 99% for E. coli, 100% for K. pneumoniae, and 91% for P. aeruginosa (112).

The concentration-time profiles of ceftazidime and avibactam, whether administered individually or in combination, exhibited consistent patterns in both single and multiple doses, irrespective of metronidazole use (**Table 1**, row a). Notably, there were no indications of time-dependent pharmacokinetics or accumulation observed in these scenarios. These findings, when combined with the outcomes of our study, propose that pairing CAZ–AVI with imipenem could serve as a viable strategy against infections caused by KPC-Kp strains, potentially revitalizing the efficacy of carbapenems (113).

**Table 1:** Four antibiotic combinations exhibit enhanced efficacy through their collective action.

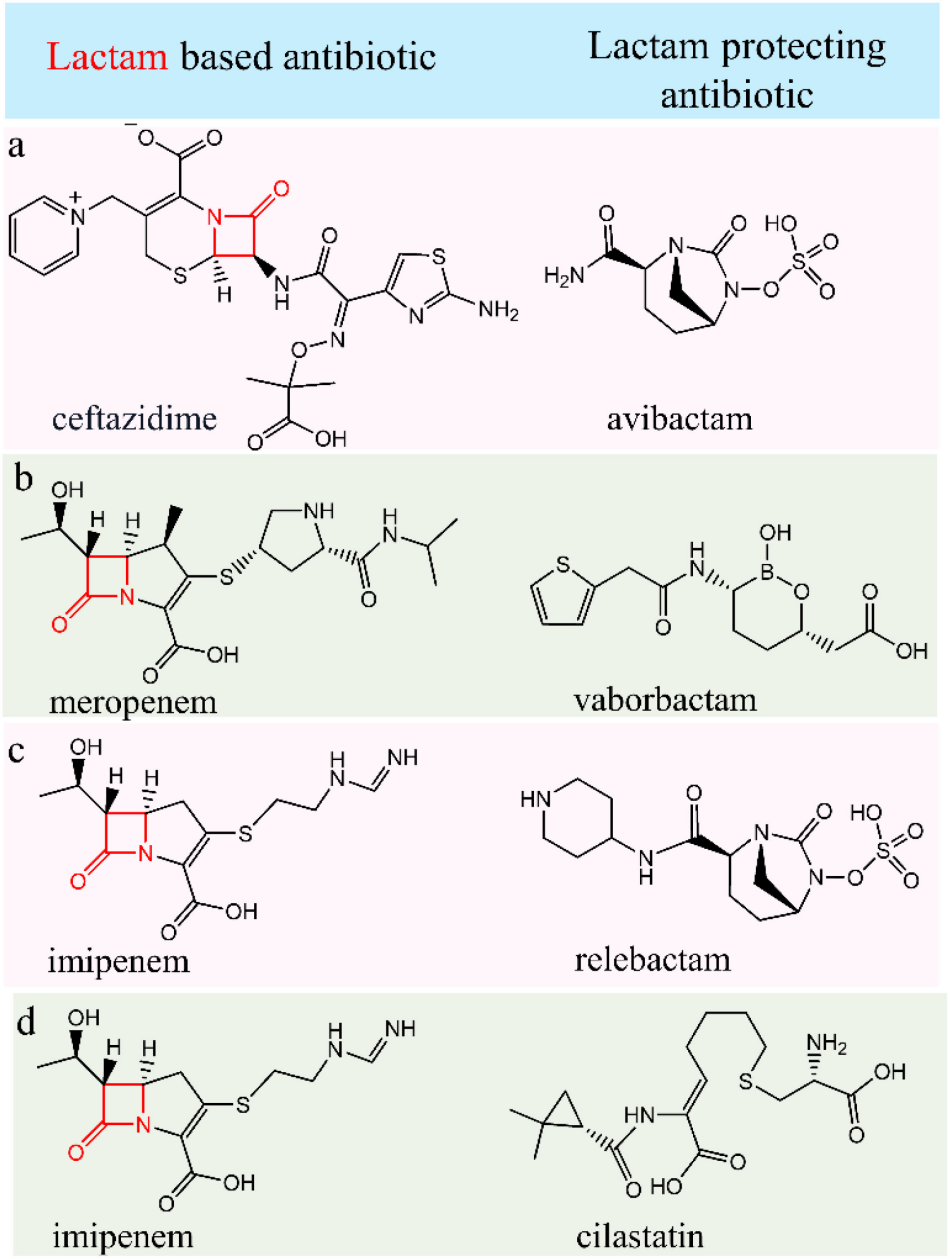	

While they may not directly synergize, one antibiotic shields another from bacterial defence mechanisms. In this table, the left-column antibiotics all feature a vulnerable lactam moiety, susceptible to bacterial attacks. Administering the second antibiotic from the right column alongside them aims to protect these lactam moieties, thereby extending their half-life and augmenting overall efficacy.

### 5.2 Meropenem/vaborbactam

Meropenem (holds a β- lactam moiety) stands as a potent broad-spectrum carbapenem antibiotic, effectively targeting both Gram-positive and Gram-negative bacteria. It targets penicillin-binding proteins (PBPs) disrupts the synthesis of crucial cell wall components, and ultimately destroys the bacterial cell. It shows remarkable binding capabilities with PBP2 and PBP3 in the Gram-negative bacteria like *Pseudomonas aeruginosa* or *Escherichia coli* and with PBP1, in the Gram-positive *Staphylococcus aureus*. Meropenem is used to treat severe infections of the skin, stomach and bacterial meningitis. On the other hand, vaborbactam is a boronic acid β-lactamase inhibitor that demonstrates a strong attraction to serine β-lactamases and Klebsiella pneumoniae carbapenemase (KPC) (**Table 1**, row b). Vaborbactam creates a reversible, covalent bond between its boronate segment and the catalytic serine found in serine β-lactamases that limits meropenem degradation. It also exhibits the strongest affinity for serine carbapenemases within Amber classes A and C (114). Thus, vaborbactam kills the bacterial β-lactamases enzymes and protects the β- lactam moiety-based meropenem. Consequently, as there is no supramolecular bonding between them, they are administered individually with a two-hour interval between doses.

Meropenem/vaborbactam (57) exhibits significant effectiveness against Enterobacterales strains that generate KPC carbapenemases, displaying slightly reduced yet noteworthy activity against strains producing MBLs or OXA-48-like enzymes (115–117). To optimize pharmacokinetic/pharmacodynamic (57) exposures and bolster bacterial eradication, a high-dose, prolonged infusion of 2 grams of meropenem and 2 grams of vaborbactam over 3 hours is administered every 8 hours. This regimen aligns with EUCAST species-related breakpoints for Enterobacterales and P. aeruginosa, designating susceptibility at 8 mg/L and resistance above 8 mg/L (118).

### 5.3 Imipenem/relebactam

Imipenem (holds a β- lactam moiety) is used to treat severe bacterial infections caused by susceptible organisms. To counteract its rapid inactivation by renal dehydropeptidase I (DHP-1), imipenem is administered alongside cilastatin, a DHP-I inhibitor. This combination enhances the half-life and tissue penetration of imipenem. Working similarly to other carbapenems, imipenem-cilastatin binds to bacterial penicillin-binding proteins, disrupting bacterial cell wall integrity and impeding synthesis (57). On the other hand, Relebactam functions as a beta-lactamase inhibitor, effectively thwarting the hydrolysis of beta-lactam antibiotics. This action significantly enhances the antibiotics’ efficacy, ensuring their increased effectiveness in combating bacterial infections (**Table 1**, row c).

### 5.4 Imipenem-Cilastatin

Cilastatin serves as a renal dehydropeptidase inhibitor, crucial for preserving the potency of imipenem by preventing its degradation. These medications are administered together to effectively address a range of infections. Imipenem and cilastatin injections are specifically employed in treating severe bacterial infections across various areas, such as abdominal, endocarditis (heart lining and valve infection), blood, respiratory, urinary, gynaecological, skin, bone, and joint infections (**Table 1**, row d). Imipenem belongs to the class of carbapenem antibiotics, known for their potent action against bacterial infections (57).

Just like these antibiotic pairs, other combinations of antibiotics follow a similar approach. One antibiotic targets a specific bacterial component, associated with the bacterial defence mechanism that causes damage to the second antibiotic. The second one, the protected antibiotic then destroys the bacteria. Examples of such antibacterial combinations include Cefoperazone/sulbactam and Ceftolozane/tazobactam (57).

While selecting the drugs from the antibiotic table for a certain bacterium, we can select the drugs in such a way that one of the two drugs shields the other by neutralizing the bacterial defence mechanism, while the protected drug eradicates the bacterial cells.

## 6 Supramolecular synthon for drug-drug supramolecular associate delivery

Supramolecular synthons refer to the precise spatial arrangement of noncovalent intermolecular interactions that construct predictable robust architecture. Crystal engineering explores supramolecular synthons for the planned design and synthesis of supramolecular materials, spanning from solid metal-organic frameworks (119), pheromone containers (120, 121) to pharmaceutical compounds (5, 6, 21, 66) and even semisolid liquid crystal gels (122) or supramolecular gels (123, 124). The precision in predicting specific supramolecular structures across solid, semisolid, and liquid crystalline states encourages the development of pharmaceutical crystals capable of maintaining stability, even within solution phases. This hypothesis gains further substantiation by observing changes in the solubility of an Active Pharmaceutical Ingredient (API) through the formation of a cocrystal in specific solvents, affirming the existence of supramolecular bonding within the solution state.

Pharmaceutical cocrystals are formed by combining specific drug molecules with a coformer for enhancing plasticity, and solubility, and reducing brittleness (21). By substituting the coformer with a complementary drug while keeping the physical parameters constant, we can improve efficacy through the formation of multidrug cocrystals. Leveraging supramolecular interactions, multiple drug molecules can unite and be jointly delivered to pathogenic cells, potentially reshaping multidrug therapy. Diverse supramolecular synthons, including acid-amide synthon, acid-pyridine dimers, acid-aminopyrimidine trimer, and carboxylic acid-amino pyrimidine synthon, hold promise for this purpose (21, 66). To develop drugs through cocrystal formation or pharmaceutical cocrystals, these formations must dissolve in water and have tablet-forming capabilities to ensure their practical use as medication.

**Figure 6:**
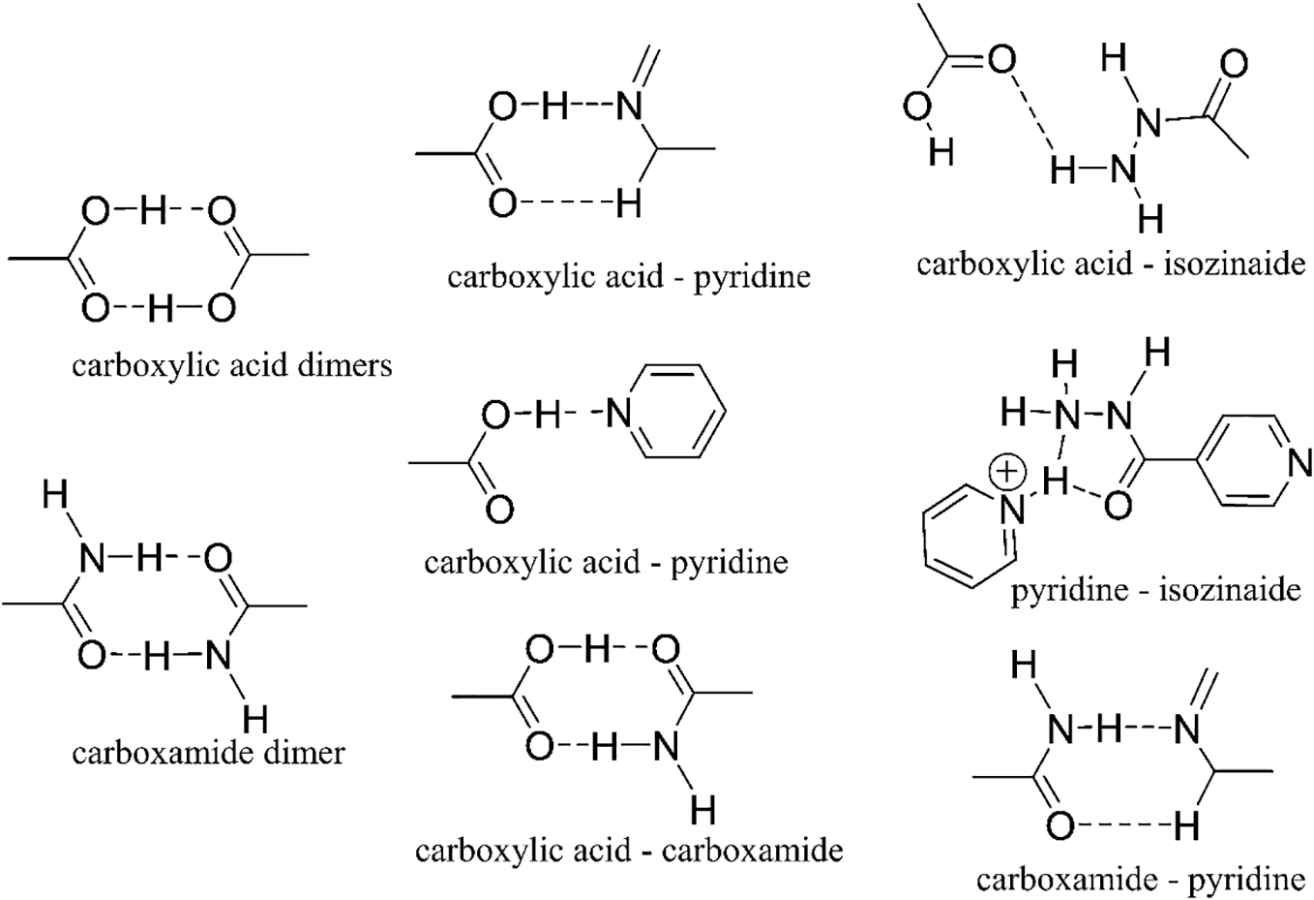
Different supramolecular synthons utilized in the development of drug-drug cocrystals can be found in the Cambridge Structure Database (CSD) (21).

To accomplish it, the crystal structure needs a 2D sheet capable of sustaining mechanical stress, suppressing brittleness, and raising the plasticity of the API. When the pharmaceutical crystal gains a twistable quality, it may display exceptional tablet-forming ability (19). Traditionally, a coformer (19) is employed in creating pharmaceutical cocrystals, which, although not a therapeutic drug molecule, are administered alongside the medicine without treating the patient’s illness. However, if a drug molecule is utilized as a coformer (19, 20) while retaining tablet-forming properties and solubility, it could significantly enhance the drug’s efficacy. **Figure 6** illustrates notable examples of extensively researched supramolecular synthons utilized in the development of cocrystals for various drug molecules including tuberculosis drugs. Cocrystal can be developed by several known techniques like slow evaporation, cooling solution, reaction cocrystallization, isothermal slurry conversion, the rapid expansion of supercritical solvents, spray drying, electrochemically induced cocrystallization, freeze drying etc (19).

### 6.1 Multidrug cocrystallization

Developing cocrystals through the combination of multiple drug molecules will soon become a prevalent technique in medicine. It can offer a simultaneous treatment for multiple diseases and also can be effective for multidrug-resistant bacteria. Although other methods such as mesoporous complexes, salts, amorphous systems, and surface-engineered nanocargos exist, cocrystals stand out due to their exceptional ability to target multiple receptors effectively and their cost-effectiveness (21).

Multidrug cocrystals (MDC) can be designed to either enhance or diminish the solubility of a specific drug. When certain drugs exhibit inadequate solubility in water, cocrystal formation proves beneficial in improving solubility. For instance, cocrystallization involving ethenzamide and gentisic acid, or Meloxicam and aspirin, has shown considerable enhancement in solubility (21). Conversely, cocrystals of lamotrigine and phenobarbital have been developed to decrease solubility, facilitating regulated drug administration (125). Another drug-drug cocrystal for glaucoma, specifically temozolomide and baicalein, demonstrates improved stability, optimized pharmacokinetics, and enhanced dissolution rates (126). However, in the case of antibiotics, it is important to deliver multiple complementary drug molecules simultaneously to the bacteria cell. Liu et al. recently showed that the tuberculosis drugs isoniazid and pyrazinamide crystallized with a bridging molecule (fumaric acid) and can be used to treat *M. Tuberculosis* (127). This marks a pioneering case of a ternary dual API cocrystal incorporating combination drugs. The cocrystal underwent assessments for aqueous solubility, dissolution, membrane permeability, and *in vivo* pharmacokinetic properties to anticipate the potential clinical efficacy of the drug. The results underscored the optimized formulation capacity of the cocrystal drug and revealed synergistic effects in both *in vitro* and *in vivo* contexts. In the last few years, people started working on cocrystalization of multiple TB drugs, however, the hypothesis behind the drug selections can make this journey effective towards the supramolecularization of PDR (128, 129). Although crystal engineering aids in identifying and formulating cocrystals, a computational approach to this process is still in development.

## 7 Principles of complementary multidrug cocrystal formation

The multidrug cocrystallization or the supramoleculization of multidrug can be based on some fundamental principles for enhancing pharmaceutical efficacy. This pioneering method targets deliver complementary drugs, better bioavailability to bacteria, and synergistic medicinal effect in the bacteria cell to maximize the therapeutic benefits. By intricately combining a variety of active pharmaceutical ingredients (APIs) with complementary drugs, multidrug cocrystallization emerges as a promising avenue for achieving synergistic effects, enhancing drug efficiency, and tailoring formulations to specific needs. This innovative approach has the potential to reshape the landscape of pharmaceutical innovation and therapeutic strategies. Once the multidrug resistance pattern is analyzed, the specific cocrystals driven from particular drugs can be administered and will work more efficiently. The multidrugs are categorized into first line, second line and third line based on their decreasing efficiency. This tiered classification assists healthcare professionals in optimizing treatment strategies based on the drug’s efficacy and tolerability.

When bacteria develop resistance to a specific drug molecule, alternative medications become necessary. Cocrystals, composed solely of multidrug-resistant bacteria treating drug molecules without external coformers, offer flexibility and tablet-forming properties, ensuring that targeted bacteria encounter multiple drug molecules. In case we can cocrystallize two or more drugs, then, even if the bacteria raise the drug resistivity, the rest drug molecules can ensure effective treatment. When formulating multidrug therapies, the selection of drug molecules should adhere to the following guiding principles.

### 7.1 Complementary drug selection

While selecting drug molecules for crystallization, they must work complementarily through various mechanisms. This synergy should result in the collateral damage of different organoids within bacterial cells. Even if a bacterium develops resistance to one drug, others should ensure its elimination. To enhance effectiveness, the incorporation of a first-line drug is recommended but not mandatory. However, their different biochemical reaction category is important to complement each other. For example, if a medicine works on a cell membrane, another should work on other organoids like DNA or mitochondria. In the case of tuberculosis, isoniazid and ethambutol exert their effects on bacterial cell walls through distinct biochemical mechanisms. Through the process of cocrystallization with rifampicin, pyrazinamide, or streptomycin, these drugs collectively target cellular organelles, expediting the destruction of bacterial cells. Pyrazinamide, given its smaller size relative to rifampicin and streptomycin, presents greater ease in the cocrystallization process. Combining complementary isoniazid, ethambutol, and pyrazinamide in a cocrystal formulation can further enhance the medication’s efficacy, potentially accelerating its action against bacterial infections.

### 7.2 Protected-protecting drug combination

After selecting the complementary drugs, we need to protect the drugs from the bacterial defence mechanism. For example, if a drug contains beta-lactam moiety, then a β-lactamase inhibitor drug should also be taken to ensure the longevity or the longer half-life period of the β-lactam-molecule. If we cocrystallize such supportive drugs behind a main drug among the complementary drug associates, then the effectivity of the multidrug therapy will be enhanced many times.

### 7.3 Implementation of supramolecular synthons

The clinical efficacy assesses how effectively the drug works for therapeutic purposes in humans. Supramolecular associates embody the remarkable potential of molecular assemblies and interactions to significantly enhance therapeutic outcomes and material properties. For designing the drug associates, we need to leverage the supramolecular synthon concept to spatially arrange and bond the different drug molecules with the maximum bonding energies and enhance the pharmaceutical parameters like solubility, plasticity, tabletability, bioactivities etc. This will evaluate the efficacy of the supramolecular system. Isoniazid and pyrazinamide show promising indications of potentially creating a carboxamide dimer or a pyridine-isoniazid synthon, as illustrated in **Figure 6**. Upon identifying the suitable components, the synthesis of cocrystals can proceed utilizing any of the methodologies elucidated at Multidrug cocrystallization section (see 6.1) section. Sometimes bridging or binder molecules can be provided to bind different drug molecules for supramolecularization of drugs (127).

### 7.4 Determination of Potency

The stoichiometric ratio would be fixed in the multidrug cocrystal, designed to be administered for multidrug-resistant bacteria. As they would work together in a complementary way, the potency of the individual molecule will not be effective, but the combination of the supramolecular drug associates should be subjected to the potency test. Potency refers to how active a drug is based on its concentration or required amount to generate a specific effect. However, as the complementary drug molecules will work synergistically, it is expected that the potency of the cocrystal will be much smaller than the individual component. Individual administration of drugs in multidrug therapy or the universal drug regimen raises the drug resistance in the MDR for their distinct mode of operation, but herein, for their synergistic effect, it’s anticipated that the potency will manifest to a lesser extent.

## 8 The efficacy of crystal engineering

Creating cocrystals using complementary drug molecules to target bacteria that tend to develop drug resistance can enhance the bactericidal effect synergistically. So far basically, coformers are incorporated into pharmaceutical cocrystals to enhance solubility (130, 131), plasticity, tabletability (132), and bioavailability (133). It also changes the melting point (134).

Yet, conformers lack medicinal properties, and substituting a conformer with a complementary drug can significantly amplify the medicinal efficacy. Therefore, the strategic design of cocrystals becomes pivotal in introducing these desirable attributes. Through the substitution of a conformer with a complementary drug molecule, cocrystals can serve diverse therapeutic purposes, including addressing different diseases or combating multidrug-resistant bacteria.

Pharmaceutical excipients play a crucial role in safely transporting drug molecules to specific organs within the body without causing any harm. The utilization of supramolecular bonding between a vital drug molecule and its complementary counterpart can obviate the necessity for pharmaceutical excipients or coformers. This dual benefit for patients involves administering complementary drugs via cocrystals while averting potential side effects associated with excipients. This approach holds significant promise, especially in treating multidrug-resistant pathogens. By implementing this method, we aim to augment the effectiveness of our treatment against bacterial infections and ultimately improve patient outcomes.

### 8.1 Supramolecular bonding for co-delivery

The co-delivery of multiple therapeutic agents holds immense potential in enhancing treatment efficacy and addressing multifaceted health challenges. Supramolecular bonding, a versatile and precise molecular assembly strategy, emerges as a promising avenue in co-delivery systems. Leveraging non-covalent interactions such as hydrogen bonding, π-π stacking, and van der Waals forces, can facilitate the formation of supramolecular drug associates, or supramolecularize the drugs, enabling tailored co-delivery formulations. Furthermore, it delves into the potential of supramolecular co-delivery systems in addressing challenges related to multidrug resistance, combination therapy, and personalized medicine. Understanding the dynamic interplay of supramolecular interactions within co-delivery systems offers a promising trajectory toward advanced therapeutic interventions, opening avenues for novel drug delivery strategies with enhanced therapeutic outcomes. All the cocrystals may not reach up to the targeted bacteria after oral administration, however, the number of bonds (135) and the strength of the bond (122) will play a very crucial role in this regard. The cocrystal can be designed by selecting two or more drug complementary drug molecules capable of forming certain supramolecular synthons. Moreover, the resultant cocrystal should be soluble in water, retaining the supramolecular association until it reaches a targeted bacteria cell. For example, when a plasmid exhibits resistance to a specific drug component within the multidrug cocrystal, the bacteria can still be targeted and killed by the second drug (21). Should the bacteria lack resistance to either or both drugs, the combined attack on the bacterial cells occurs simultaneously. The innovative use of supramolecular bonds to merge pharmaceutical molecules, enabling a unified assault against bacteria, represents a novel approach. This method significantly enhances the efficacy of multidrug therapy, restricting bacteria from rising resistance to the synergistic attack (136). In the context of multidrug-resistant bacteria, alternative supramolecular materials utilized for drug delivery, such as supramolecular gels (137, 138) and metal-organic frameworks (MOFs) (139, 140), might not exhibit the same precision in targeting bacterial cells as cocrystals do. This discrepancy arises from the fact that encapsulated or trapped drug molecules within these materials might not readily present the essential functional groups to initiate an immediate reaction with the bacteria. Despite the ongoing efforts to prepare drug-drug cocrystals, the proactive development of complementary drug molecules for synergistically combating multidrug-resistant bacteria is yet to commence (141–143).

### 8.2 Harmonization of complementary cocrystal therapy

Pharmaceutical harmonization aligns with global regulations, ensuring consistency in drug development, manufacturing, and quality control. This collaboration streamlines approvals, supports trade, and enforces universal safety and efficacy standards. Developing a new regimen of 3-4 drugs for multidrug therapy usually spans 15-20 years. However, once the drugs are approved for multidrug therapy, can be considered quickly for developing the cocrystals. The present momentum behind multidrug therapy regimens is accelerating through effective collaboration among upcoming generations of drug trial researchers, controllers, and policymakers (143, 144).

A minor portion of antibiotics approved in the last four decades introduces new compound classes. Instead, the majority stem from existing chemical structures, with the most recent antibiotic class discovered in the 1980s (145). As developing a new antibiotic is a tedious job, M. Miethke et al. suggested the formation of an international coalition comprising seasoned antimicrobial resistance (AMR) lobbyists (146). This group would collectively advocate for funding aimed at early antibacterial drug discovery research, aligning with the principles outlined in this article.

The progress of innovative strategies behind tuberculosis (TB) therapeutics (147, 148) is presently gaining momentum through the harmonization of TB drug trial researchers, manufacturing, controllers, and policymakers (144). In the last 5-10 years a movement is started in developing the universal drug regimen (pan-TB regimens), due to the expanding pipeline of anti-TB drugs with distinct modes of action. Harmonization reduces the time to bring a new drug regimen to the market. Once a PAN regimen is developed, it can also address the lower drug-resistant bacteria categories like extensively drug-resistant (XDR), multi-drug-resistant (MDR), and drug-susceptible *M. tuberculosis* infections. The potential for universal regimens to expedite global TB control is considerable. Still predicting the finest regimens presents a challenge due to the numerous potential combinations involving three, four, or five drugs across ten essential drug classes (74). Presently, the ranking of regimens is affected by restricted preclinical data, highlighting the need for more robust clinical trials to enhance this assessment process (149). Similar to the development process effective binary combination of two antibiotics, a clinical is going on here. The discovery of effective combinations remains serendipitous. To address this issue, a complementary combination with drug-drug interaction may facilitate and accelerate the effectiveness of the antibiotic combination process. The drug-drug interaction or supramolecular assembling of drugs or forming the cocrystals that are bonded strongly even inside the biological system can deliver the drug combination to the targeted bacteria. Then after determining the potency, the harmonization would accelerate bringing the drugs into the market. The very similar efforts are also needed for every drug-resistant bacterium (150).

## 9 Advantages of complementary cocrystal therapy

Conventional multi-drug therapy administers drugs separately, leading to individual drug molecule administration and boosting drug resistance. By forming bonds to deliver these drugs simultaneously to targeted bacterial cells, we can achieve several advantages.

### 9.1 Minimal drug-drug interactions

The drug-drug interactions often exhibit unwanted side effects. However, herein, developing drug-drug interactions through forming cocrystals and administering them in the body will minimize the unwanted side effects.

### 9.2 Minimizing bacterial drug resistance

Unlike multidrug therapy or universal drug regimen therapy, this process promotes working the complementary drug molecule together to a targeted multidrug-resistant bacterium cell and minimizes the risk of raising drug resistance.

### 9.3 Minimizing side effects

This process will minimize the consumption of unnecessary medicines and will help in reducing the side effects. For their synergistic work inside a bacterium cell, the multidrug-based cocrystal therapy will minimize the treatment time, cost, mortality and probability of spreading the disease in society.

### 9.4 Exact potency determination

We can determine the exact potency for the multidrug therapy which was not possible in multidrug therapy.

### 9.5 Antibacterial coating

Antibacterial coatings represent an innovative approach in various sectors, from healthcare to consumer goods. These coatings are designed to inhibit the growth of bacteria on surfaces, thereby reducing the risk of infections and enhancing overall hygiene. Composed of antimicrobial agents or materials, they act as a protective layer, hindering the colonization and proliferation of bacteria. In healthcare settings, antibacterial coatings on medical devices, such as catheters or implants, help prevent healthcare-associated infections. As hospital walls and furniture stay infected by various highly immune bacteria, a proper coating is mandatory to prevent the spreading of infection. Coating the surfaces of ceilings, chairs, doors, tables, walls, windows, and furniture with the cocrystal will reduce the risk of spreading MDR bacteria in hospitals or the patient’s home. As cocrystals are hard to stick over the surface, they can be doped in some antibacterial polymers (151) or can be mixed with the dye (152), sonocoating methods (153). or by spray drying (19). Once we can coat the surface of the furniture or wall by polymer with the free -COOH group like an oxidized carbon nanotube (154), then we can coat the surface with an amine-containing group or vice versa.

## 10 Limitation of complementary cocrystal therapy

Though it is supposed to kill the multidrug-resistant bacteria much faster in comparison to the other conventional therapies, still during the treatment it may show drug resistivity up to a certain extent. Experimental studies can show the exact efficacy of this therapy.

However, we may face also some challenges behind the drug formulation. The establishment of supramolecular bonds (155–158) between complementary drug molecules or within a ‘protecting-protective’ combination isn’t always achievable. Those targeted two or more drugs might be difficult to crystallize together. Moreover, forming certain supramolecular synthons, some more technical difficulties may arise in co-delivering the supramolecular multidrug.

Supramolecular limitations

i) Creating supramolecular synthons presents challenges, particularly in delivering them to bacterial cells. The existence of multiple competitive synthon formation probabilities often can end up with some unpredicted crystal structures with enhanced or reduced bio-activities. However, still they can be harnessed for cocrystal-based multidrug therapies.
ii) If the number of supramolecular bonds and the bond energy amounts are too high, then the cocrystals can be difficult to use for multidrug therapy. For example, the cocrystals made of Melamine and Cyanuric Acid are so much stable, that they are stable enough in the highly acidic environment under the electrochemical environment (135). Such strong bonding won’t be soluble in water and can never be used for administering the drug molecules.
iii) Ammonium carboxylate salts, supported by charge assistance and featuring a Δ pKa value below 3.5, can render the cocrystal unstable in the presence of any protic solvent due to the high solubility of any individual component (120, 121, 159). Additionally, instability may arise if one of the individual components possesses high solubility and exhibits weak supramolecular bonding in its cocrystal form. By exploiting the Δ pKa value difference, pheromone can be released from the supramolecular container or the cocrystal to release the major pheromone to control the pests (121, 159). The lability of the proton at the ammonium carboxylate bonding in this salt allows the pheromones to be freely released into the atmosphere due to the trace amount of water (acting as a catalyst) present.

## 11 Bacteria wise remedy

Nanomarkers represent a breakthrough in precision medicine, offering a glimpse into the efficacy of drugs against specific bacteria. These markers delve into the genetic intricacies of microbial populations, enabling the identification of drug-resistant strains. By analyzing biomarkers, nanomarkers can decipher the genetic signatures associated with drug resistance mechanisms within bacteria (160). This technology allows for swift and accurate assessments of drug effectiveness, aiding healthcare professionals in tailoring treatment regimens to combat resistant infections. The ability to discern which drugs may be rendered ineffective against certain bacterial strains before administering treatment holds tremendous promise in optimizing patient care and combating antibiotic resistance (161). Nanomarkers stand as a pioneering tool in the fight against drug-resistant bacteria, revolutionizing how we approach infectious disease management. Thus, after analyzing the drug resistance ability, once we can administer the multidrug cocrystal accordingly, we can protect the MDR bacteria very quickly.

## 12 Conclusion

Leveraging AI expedites the development of antibiotics, streamlining the process by utilizing advanced technologies. This will help synthesize the active antibiotic molecules faster and provide more options to develop the drug regimens faster. Through the binary antibacterial combinations, that involve the strategic pairing of two antibiotics: one serves as a protective shield, safeguarding the other, while the second antibiotic works actively to neutralize harmful microbial molecules. This tandem approach enhances the overall effectiveness of the treatment against resistant bacteria.

With such an ongoing process, we need to introduce the administering of the multidrug together, so that bacteria won’t get the required time to raise their defense mechanism. Crystal engineering introduces a novel dimension to this approach by allowing the creation of multidrug-driven cocrystals. These cocrystals can be formed by pairing two or more complementary drug molecules or by combining protected-protected antibiotic combinations. This innovative strategy targets multi-drug-resistant bacteria with heightened precision and efficacy. Determining the potency of these cocrystals becomes crucial, as it paves the way for a proactive multi-drug approach. This proactive method harnesses the synergistic effect of the combined antibiotics, effectively eliminating bacteria while preventing the escalation of their defence mechanisms.

This advanced approach not only accelerates the multidrug therapy process but also ensures a more thorough and rapid eradication of bacterial infections, marking a significant leap forward in combating antibiotic-resistant strains.

## Data availability statement

The original contributions presented in the study are included in the article. Further inquiries can be directed to the corresponding author.

## Author contributions

PS: Conceptualization, Methodology, Software, Writing – original draft, Writing – review & editing.
